# Single-cell duplex RT-LATE-PCR reveals *Oct4 *and *Xist *RNA gradients in 8-cell embryos

**DOI:** 10.1186/1472-6750-7-87

**Published:** 2007-12-07

**Authors:** Cristina Hartshorn, Judith J Eckert, Odelya Hartung, Lawrence J Wangh

**Affiliations:** 1 Department of Biology, Brandeis University, Waltham MA 02454-9110, USA; 2 DOHaD Division, School of Medicine, Developmental Cell Biology Group, School, of Biological Sciences, University of Southampton, Bassett Crescent East, Southampton, SO16 7PX, UK

## Abstract

**Background:**

The formation of two distinctive cell lineages in preimplantation mouse embryos is characterized by differential gene expression. The cells of the inner cell mass are pluripotent and express high levels of *Oct4 *mRNA, which is down-regulated in the surrounding trophectoderm. In contrast, the trophectoderm of female embryos contains *Xist *mRNA, which is absent from cells of the inner mass. Prior to blastocyst formation, all blastomeres of female embryos still express both of these RNAs. We, thus, postulated that simultaneous quantification of *Oct4 *and *Xist *transcripts in individual blastomeres at the 8-cell stage could be informative as to their subsequent fate. Testing this hypothesis, however, presented numerous technical challenges. We overcame these difficulties by combining PurAmp, a single-tube method for RNA preparation and quantification, with LATE-PCR, an advanced form of asymmetric PCR.

**Results:**

We constructed a duplex RT-LATE-PCR assay for real-time measurement of *Oct4 *and *Xist *templates and confirmed its specificity and quantitative accuracy with different methods. We then undertook analysis of sets of blastomeres isolated from embryos at the 8-cell stage. At this stage, all cells in the embryo are still pluripotent and morphologically equivalent. Our results demonstrate, however, that both *Oct4 *and *Xist *RNA levels vary in individual blastomeres comprising the same embryo, with some cells having particularly elevated levels of either transcript. Analysis of multiple embryos also shows that *Xist *and *Oct4 *expression levels are not correlated at the 8-cell stage, although transcription of both genes is up-regulated at this time in development. In addition, comparison of data from males and females allowed us to determine that the efficiency of the *Oct4*/*Xist *assay is unaffected by sex-related differences in gene expression.

**Conclusion:**

This paper describes the first example of multiplex RT-LATE-PCR and its utility, when combined with PurAmp sample preparation, for quantitative analysis of transcript levels in single cells. With this technique, copy numbers of different RNAs can be accurately measured independently from their relative abundance in a cell, a goal that cannot be achieved using symmetric PCR. The technique illustrated in this work is relevant to a wide array of applications, such as stem cell and cancer cell analysis and preimplantation genetic diagnostics.

## Background

Accurate quantification of multiple target sequences by real-time polymerase chain reaction (PCR) has been proven difficult to achieve, particularly for measuring numbers of RNA transcripts, rather than of DNA copies [1]. In fact, while different gene sequences are represented in the genome in similar numbers (one or two copies, depending on the chromosomal location and the possible presence of mutations), transcript levels of different genes can vary widely. Moreover, changes in gene expression are often rapid and transient in response to stimuli, stress, or cellular events such as cell division and cell differentiation. In order to detect meaningful variations in transcript numbers it is, thus, necessary to measure them in series of single cells rather than in cell cohorts where individual differences could be lost to "background noise [2]." From all of the above considerations it follows that a convenient and reliable experimental approach, sensitive enough to measure RNA copy numbers in individual cells, is essential for multiplex quantification of gene expression in biological systems.

We have recently developed an entirely single-tube method to measure mRNA levels in individual cells ("PurAmp") [3]. For that and a previous study [4] we co-amplified and simultaneously quantified RNA and DNA copies of the *Xist *and the *Sry *genes in mouse embryos and blastomeres. These two genes were chosen because they have sexually distinct patterns of expression in the early embryo. Female cells contain two copies of the *Xist *gene, one on each X-chromosome, and high levels of *Xist *transcripts but lack the *Sry*-bearing Y-chromosome, while cells from early-stage male embryos have a single unexpressed copy of the *Xist *gene on the X-chromosome and a single unexpressed copy of *Sry *on the Y-chromosome. Hence, only *Xist *templates (cDNA+ genomic DNA) were amplified from female samples, while male embryos always generated equal numbers of *Xist *and *Sry *amplicons.

In these studies we were, therefore, never faced with the more common and problematic situation of having to simultaneously quantify unequal amounts of different target sequences, a technical challenge of great general interest. In fact, conventional symmetric PCR is not easily utilized in this situation due to the exponential nature of the reaction. With this method, abundant templates generate amplicons much more rapidly than scarcer templates and the reaction is progressively shut down by these early-accumulating double-stranded molecules that sequester the DNA polymerase, and by a number of other factors [5]. In the case of real-time PCR the fluorescent signal of the most abundant amplicon will reach its threshold cycle (or C_T _value, used to quantify template copy numbers [6]) and plateau unaffected by the presence of any other less represented template. Amplification of the less numerous templates, on the other hand, will be greatly hindered [5]. Thus, low copy number templates will either go undetected or their abundance will be under-estimated based on their delayed C_T _values.

Linear-After-The-Exponential (LATE)-PCR, invented in our laboratory, provides a much more reliable strategy for the quantification of multiplexed templates. This approach combines the efficiency of exponential amplification in the very early phases of the reaction with the advantages of linear amplification [7,8]. The switch between the exponential and the linear phases of LATE-PCR occurs at the C_T_, independently for each template, so that template quantification is achieved exactly as in symmetric PCR. After the C_T _is reached, a single strand of each amplicon continues to accumulate linearly and, thus, at a rate much slower than in symmetric PCR. This feature and the fact that single-stranded amplicons do not engage the polymerase ensure efficient amplification of abundant as well as rare templates in a sample. Moreover, single-stranded amplicons afford great flexibility for the design of specific probes because the probe is free to bind to the amplicon without having to compete with a complementary strand. Single-stranded LATE-PCR products can also be easily and directly sequenced to confirm their identity and purity ([9]).

As reported here, the properties of LATE-PCR have enabled us to expand our earlier studies on differential gene expression in blastomeres of preimplantation mouse embryos [4,10,11]. We focus in particular on the 8-cell stage embryo prior to compaction, when individual blastomeres can still be dissected and collected with a limited amount of manipulation. This developmental stage immediately precedes the formation of an embryo comprised by inner and outer cells containing distinct sets of proteins in response to their different surroundings [12-14]. It is therefore plausible to hypothesize that differences in gene expression between blastomeres of early 8-cell embryos may presage subsequent cell fate and, thus, may identify the founders of the cell lineages established during the last phase of preimplantation, the blastocyst stage [15,16].

*Xist *RNA is an untranslated transcript responsible for X-chromosome silencing and dosage compensation in female cells. Its expression precedes X-chromosome inactivation and *Xist *RNA is present in cleavage-stage embryonic blastomeres [17,18]. In mouse blastocysts, *Xist *expression is paternally-imprinted; *Xist *RNA is restricted to the cells of the trophectoderm (TE) surrounding the embryo and destined to generate extraembryonic tissues such as the placenta, while it is almost absent from the pluripotent inner cell mass (ICM) that gives origin to the embryo proper [19,20]. The gene for the transcription factor Oct4 presents a reciprocal expression pattern, being highly transcribed in the ICM and down-regulated in the TE [19,21,22]. Initial studies on cleaving embryos have shown that the Oct4 protein is ubiquitously present in the blastomeres starting at the 4-cell stage [21,22], but cell-to-cell differences were not assessed. More recently, the importance of quantitative data has become increasingly clear because expression of the *Oct4 *gene (also previously indicated as *Pou5f1*or *Oct3/4*) is necessary to maintain cell pluripotency via a stringently dose-dependent regulatory process. Variations in Oct4 levels above or below the required dosage produce cellular differentiation [23] and usually consist in this gene's repression concomitant to activation of lineage- or tissue-specific genes [22,24,25]. Hence, cells maintaining critical amounts of *Oct4 *RNA during early development are candidate to be ICM precursors, while lower *Oct4 *RNA levels and elevated *Xist *expression may indicate a trophectodermal fate. We and others have already reported that blastomeres of mouse 8-cell embryos contain different amounts of *Xist *RNA [4,26]. Non-quantitative analyses of human cleavage-stage blastomeres have also shown a variable distribution of *Oct4 *RNA [27,28], but, for ethical reasons, the cells available for these studies are limited in number and quality. To more thoroughly address the question of cell lineage onset, in the present work we simultaneously measured *Xist *and *Oct4 *RNA levels in series of blastomeres from male and female mouse embryos at the 8-cell stage. The results presented in this paper are the first report of quantification of cDNA with LATE-PCR, and demonstrate the utility of this technique for gene expression studies, particularly at the single-cell level where multiplexing targets is highly desirable.

## Results

### Specificity of the *Oct4 *LATE-PCR assay and identification of *Oct4 *pseudogenes

As detailed elsewhere [3,4,10], our approach is to co-amplify both the cDNA and genomic DNA of each gene under study by designing PCR primers within one of the gene's exons. Single cells or embryos are lysed directly into a PCR tube and both DNA and RNA molecules are released and de-proteinized in the same, single vessel where reverse transcription (RT) and PCR are also carried out. This strategy optimizes the accuracy of quantification and offers three main advantages. First, it allows the use of genomic DNA for standardization, ensuring that PCR efficiency will be exactly the same for the standard and the samples analyzed. Second, it makes it possible to count DNA copy numbers in the absence of RT or in cells or embryos that do not express the gene of interest (such as *Xist *in male cells, see below). The presence of the genomic sequence in the sample tells us that the cell has been successfully transferred to the assay tube, even in the absence of RNA. This feature is also useful to identify RNase contamination problems if quantitative PCR analysis demonstrates the presence of the expected gene sequence but not of its transcripts. Third, co-amplification of genomic DNA and cDNA (representative of RNA) eliminates the need for DNase digestion, a step known to affect RNA recovery.

Construction of a quantitative *Oct4 *DNA-specific PCR assay presented a number of technical challenges because this gene is part of a highly homologous and conserved family of transcription factors [29]. *Oct4 *appears to be the most actively transcribed gene of this group during early mammalian embryogenesis, decreasing the concern for non-specific amplification of homologous RNAs, although some *Oct1 *and *Oct6 *expression has also been reported [29-33]. At the DNA level, however, the concern remains, compounded by the high frequency of retrotransposition observed for embryonic stem cell-specific genes including *Oct4 *[34]. Additionally, the *Oct4 *sequence is highly GC-rich, which favors secondary structure and requires very stringent parameters to ensure specific but efficient amplification, particularly when a second primer pair and two probes are introduced in a duplex reaction. By running a BLAST search we identified sequences highly homologous to *Oct4 *mRNA on mouse chromosome 1 (GenBank Accession Number AC126050.3) and chromosome 3 (GenBank Accession Number AC107368.11). The presence of these previously unreported pseudogenes was not surprising, based on similar findings in the human and bovine genomes [34,35]. Based on this knowledge and avoiding sequences repeated in the mouse genome, we constructed an *Oct4*-specific LATE-PCR assay taking advantage of the flexibility that this technique affords for primer design [8]. A longer, high T_m _primer (the limiting primer) was designed from a very GC-rich sequence in order to include several nucleotides different from the most homologous pseudogene, while a shorter, lower T_m _primer (the excess primer) could be selected in an area of the gene less homologous to other DNA sequences. (See Methods for details on the LATE-PCR assay and conditions.)

### Validation of the *Oct4 *RT-LATE-PCR assay in a biological system and by sequencing

**Figure 1:**
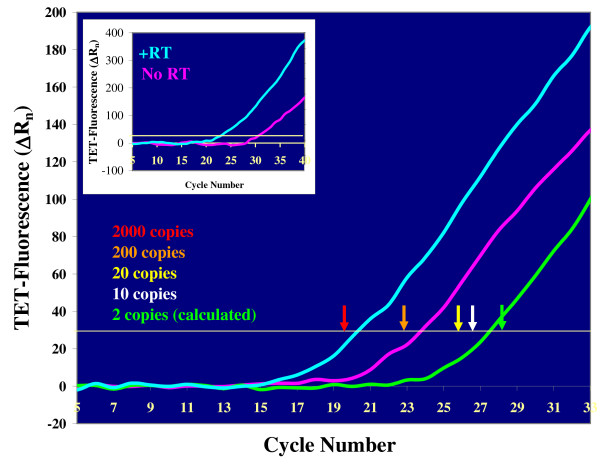
**Biological validation of the *Oct4 *RT-LATE-PCR assay: *Oct4 *expression in the two lineages of blastocysts**. Real-time LATE-PCR plots generated by *Oct4 *templates in the following samples: expanded blastocyst after RT (blue line, RNA + DNA), expanded blastocyst without RT (pink line, DNA only) and single TE cell after RT (green line, RNA + DNA). Whole blastocysts contain *Oct4 *RNA (as shown by the delta between the pink and the blue line) due to the presence of the ICM. *Oct4 *expression is downregulated in the TE (the green line crosses the threshold very close to the 2-copy marker; two *Oct4 *template copies per cell are due to the presence of *Oct4 *genomic DNA). The positions of the white-to-red arrows correspond to known copy numbers in a genomic DNA standard, as color-coded; the placement of the green arrow was calculated based on this standard. Inset: *Oct4 *expression in the ICM isolated from an early blastocyst. Half of the ICM was used for the "+RT" determination (blue line) and half was used for the "No RT" determination (pink line). The probe used in all these experiments was an *Oct4*-specific molecular beacon conjugated to TET.

*Oct4 *expression was initially analyzed in individual embryos at the late blastocyst stage, as illustrated by the examples in Fig. 1. The blue line shows the *Oct4 *amplification plot generated by a single blastocyst that had undergone RT before PCR and, therefore, contained both genomic DNA and cDNA. The pink line, in contrast, shows the *Oct4 *amplification plot generated by a single blastocyst that did not undergo RT before PCR and, therefore, contained only genomic DNA. Serial dilutions of purified genomic DNA were assayed in parallel with the embryos and used to provide a standard for quantification of the *Oct4 *template copy numbers. The C_T _values of the standards are shown in the figure by color-coded arrows and reflect the fact that each genome contains two copies of the *Oct4 *gene. The blue line crosses the threshold one cycle after the 2000-copy marker, indicating that this sample contained 1000 total *Oct4 *templates (genomic DNA + cDNA). The copy number calculated from the C_T _value of the pink line (genomic DNA only) was 100, corresponding to 50 cells (based on the assumption of two copies per cell) [3,4,10,18]. The template number difference between the "+RT" and the "No RT" samples confirmed active *Oct4 *expression (900 copies of *Oct4 *RNA), as expected in blastocyst-stage embryos [21,22].

As a next step, we separately analyzed *Oct4 *expression in each of the two lineages present in blastocyst stage embryos. Single TE cells or small groups of TE cells protruding from the zona pellucida in blastocysts that had initiated hatching could be removed from the embryo and were tested for their *Oct4 *RNA + DNA content. The green line in Fig. 1 shows the amplification plot obtained from a single TE cell after RT-PCR. This signal was converted to only four template copies, at least two of which are genomic DNA, consistent with the expected down-regulation of *Oct4 *expression in TE cells. The additional two copies may be residual *Oct4 *RNA or genomic DNA, if the TE cell had completed DNA duplication. We then measured *Oct4 *expression in ICMs extracted from blastocysts by immunosurgery. The ICM isolated from each blastocyst was divided in two aliquots, one processed by RT-PCR (blue line in the inset of Fig. 1) and one by PCR only (pink line in the inset of Fig. 1). Immunosurgery requires the use of embryos at the very early blastocyst stage; for this reason, the *Oct4 *RNA levels found in these ICMs cannot be directly compared to those measured in the mature blastocysts. However, the ΔC_T _between the pink and the blue line is larger for the isolated ICM (six cycles, inset of Fig. 1) than for the whole blastocysts (three cycles, Fig. 1), signaling the presence of a higher *Oct*4 RNA copy number per cell in the ICM. ICM cells comprise only about one-third of the blastocyst's cells, thus lower levels of *Oct4 *transcripts per cell in whole blastocysts than in isolated ICMs are consistent with the expected active *Oct4 *expression in the cells of the inner mass and down-regulation in the TE. Taken together, these quantitative data provided a validation of the specificity of our LATE-PCR assay in a known biological system.

We further confirmed by sequencing the specificity of the *Oct4 *amplicons generated from either "+RT" or "No RT" samples prepared from 8-cell embryos. As expected, both genomic DNA and cDNA amplicons had the same sequence, identical to that of the *Oct4 *gene at the designated amplification site (GenBank Accession Number AH003838). Sequencing of both the "+RT" and "No RT" amplicons confirmed that the nucleotide in position 312 of this sequence (segment 1) is a C, and not a T as reported in the *Oct4 *mRNA sequence that we had used to design the primers and the probe (GenBank Accession Number NM_013633, residue 140). Since neither primers nor probe included this residue, PCR results are unaffected.

### Construction and optimization of a duplex LATE-PCR assay for simultaneous quantification of *Oct4 *and *Xist *templates

**Figure 2:**
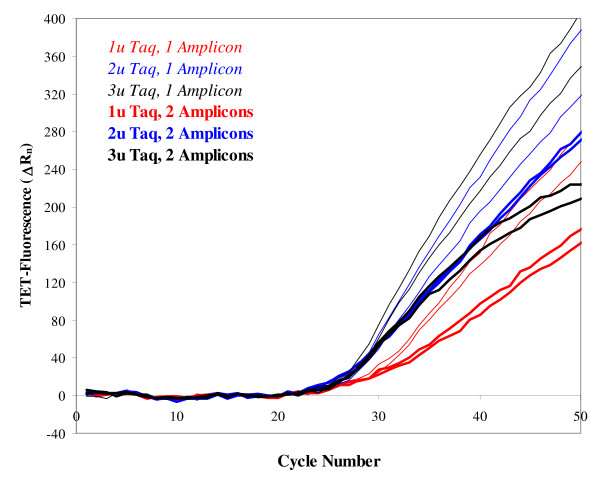
**Optimal DNA-polymerase concentration for amplification of *Oct4 *templates in single-amplicon vs duplex LATE-PCR assays**. Fluorescent signals generated by *Oct4 *DNA under different amplification conditions. One-thousand mouse genome equivalents, containing 2000 copies of *Oct4 *DNA, were used for each assay; all tests were run in duplicates. For this experiment, the PCR profile described in the "Methods" section was slightly modified in order to highlight the Taq-polymerase concentration effect, shortening the second stage from 15 to 6 cycles, but similar results were obtained with either of the two PCR profiles. In one group of samples *Oct4 *was the only template amplified (shown by the thin lines). In a second group of samples *Oct4 *(shown by the thick lines) was co-amplified with *Xist *(not shown) by addition of *Xist*-specific primers. Increasing amounts of Taq polymerase were added to the samples in each group, as follows: red lines, 1 u per assay; blue lines, 2 u per assay; black lines, 3 u per assay. The presence of Taq at the highest concentration and of a second set of primers favors non-specific amplification with a negative effect on *Oct4 *amplicon production, as shown by the declining slope of the thick black lines. Thus, 2 u of Taq polymerase per assay represent the optimal enzyme concentration in the duplex, resulting in specific amplification with maximal efficiency (steepest slope).

Co-amplification of *Xist *templates together with the *Oct4 *templates described above was achieved following the same strategy. However, the introduction of a second pair of primers in the reaction required a second level of optimization of the assay, which took in account possible new, non-specific interactions involving the additional oligonucleotides as well as the higher rate at which Taq polymerase is used in a multiplex PCR. We found that the optimal concentration of Taq polymerase to be used for LATE-PCR assays can be established by analysis of the real-time amplification plots. The *Oct4 *amplification plots generated by the same amount of genomic DNA in the presence of different Taq polymerase concentrations are reported in Fig. 2. The thin lines were obtained from amplification of *Oct4 *templates only, while the thick lines show amplification of *Oct4 *in the duplex assay. (In the latter case, *Xist *amplification occurred in parallel but is not reported.) When only one pair of primers was present (thin lines), increasing Taq concentration from 1 (red) to 2 (blue) or 3 (black) units per assay had the effect of making the slope of the signals steeper, due to increased amplification efficiency. The signals produced in the presence of 2 and 3 units per assay were interdispersed, suggesting that maximal efficiency had been reached around this level. As expected, the slopes of the lines generated during the duplex reactions (thick lines) were in all cases lower than those generated by amplification of a single amplicon, because the Taq polymerase was used at twice that rate. As in the case of the single-amplicon LATE-PCR, augmenting Taq concentration in the duplex reaction from 1 to 2 or 3 units per assay resulted in an increase of the signals' slopes. There was no further increase in the initial slope of the 3 Taq-unit group when compared to the initial slope of the 2 Taq-unit group, again suggesting that maximal efficiency had been reached. However, the 3 Taq-unit group quickly reached a plateau and its slope started declining, unlike that one of the 2 Taq-unit group. This occurrence suggests that the highest Taq concentration tested promoted mis-priming. Mis-priming leads to co-amplification of non-specific targets together with the desired amplicon. Because the probe is designed to selectively bind to the intended template only, fluorescent signals decline in case of non-specific amplification. It is interesting to notice that this did not happen when the samples still contained 3 units of Taq but only one pair of primers (compare thick and thin black lines). In spite of the higher amount of available Taq in the single-amplicon assays when compared to the duplexes (3 units being used to generate one amplicon rather than two amplicons at the same time), more mis-priming occurred in the duplexes due to the addition of the *Xist *primers. In a similar experiment (not shown), these results were confirmed by agarose gel analysis of the amplification products of the duplex assay in the presence of 2 units of Taq (specific amplicons only) or 3 units of Taq (a non-specific amplicon was also present).

**Figure 3:**
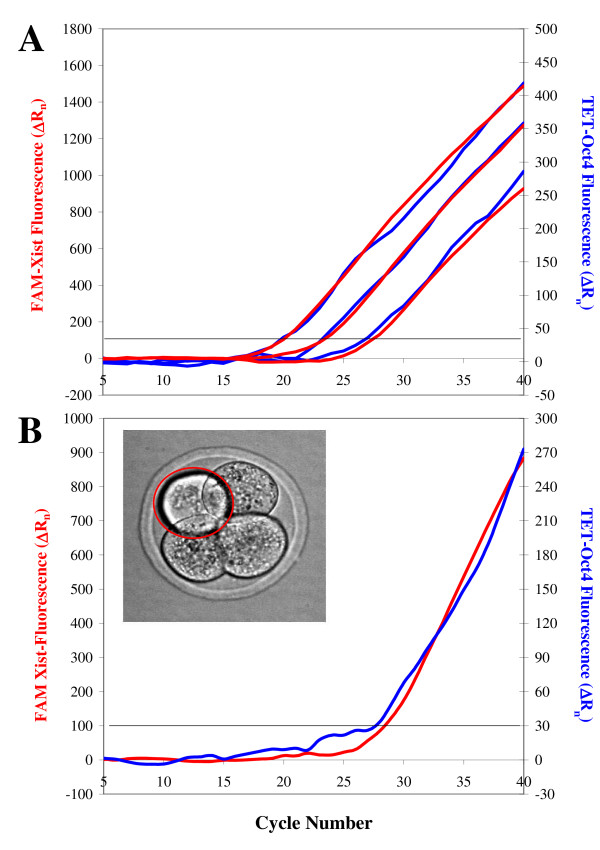
**Simultaneous amplification of *Oct4 *and *Xist *genomic DNA even at low template copy numbers**. A FAM-conjugated molecular beacon was used to monitor *Xist *amplification (red lines; fluorescence scale on the left of each graph) and a TET-conjugated molecular beacon was designed for *Oct4 *template quantification (blue lines; fluorescence scale on the right of each graph) in a duplex LATE-PCR assay. (A) Serial dilutions of male mouse DNA, containing respectively (left-to-right) 1000 genomes, 100 genomes and 10 genomes, show consistent co-amplification of *Oct4 *and *Xist *DNA at all three dilutions tested. The C_T _values of standard scales such as this were used for template copy number quantification in unknown samples. Fluorescent signals arising earlier (smaller C_T _values) indicated the presence of more templates than later-arising signals. (B) *Oct4*/*Xist *LATE-PCR analysis of a single blastomere isolated from a 4-cell stage embryo. The real-time fluorescent signals reveal the presence of both *Oct4 *DNA (known to be present in two copies per cell) and *Xist *DNA (one copy in male cells, two copies in female cells). RT was not performed in this experiment, thus the sex of this embryo could not be determined by *Xist *RNA quantification.

The above data demonstrate that the concentration of Taq polymerase needs to be optimized based both on the number and the sequences of the primers added to a multiplex reaction, in order to obtain maximal efficiency without mis-priming. In addition, they illustrate the diagnostic potential of the slopes from LATE-PCR real-time assays. These slopes are the result of linear amplification and are, therefore, much more sensitive to changes in PCR efficiency than symmetric PCR slopes. We have found that in suboptimal conditions, when one of the amplicons in a duplex is generated more efficiently than the other, the slope of the weaker amplicon changes depending on the presence or absence as well as on the abundance of the first, "stronger" template. Taking advantage of this characteristic, we have titrated PrimeSafe-043, a reagent that prevents mis-priming (see Methods) in order to obtain the best amplification conditions for both *Oct4 *and *Xist *amplicons, independent of their relative abundance. The real-time PCR plots generated by serial dilutions of standard genomic DNA in the optimized duplex assay are reported in Fig. 3A. At the appropriate fluorescence scale for each of the two molecular beacons used, the *Xist *(red) and *Oct4 *(blue) amplification curves coincided, simplifying the process of C_T_-based quantification of unknown samples. In addition, different amounts of templates at the beginning of the reaction produced proportionally different levels of final (end-point) fluorescence. The sensitivity of this assay was proven by co-amplification of *Oct4 *and *Xist *genomic DNA from single blastomeres (Fig. 3B); for these "No RT" experiments, PCR was performed directly after cell lysis.

### *Oct4 *and *Xist *RNA + DNA measurements in series of blastomeres of male and female 8-cell embryos

**Figure 4:**
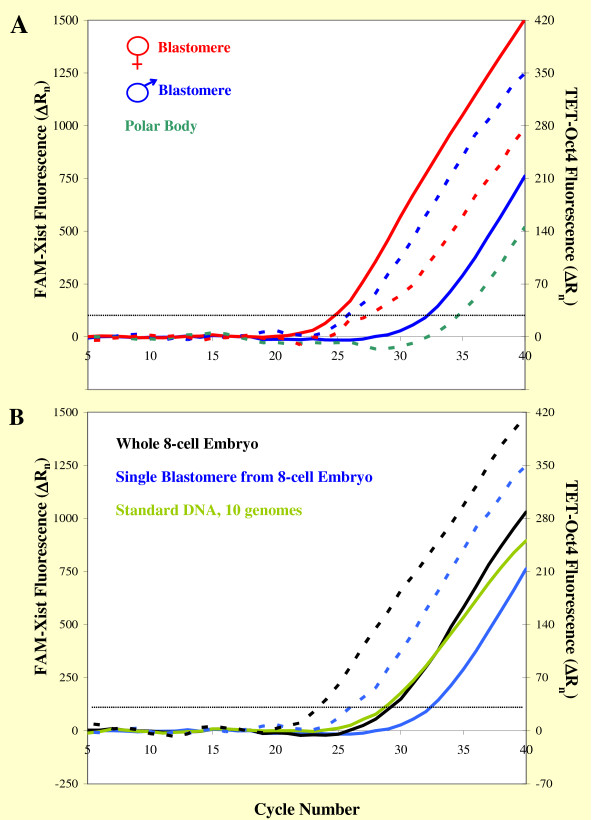
**Quantitative analysis of *Oct4*/*Xist *RNA + DNA templates in single blastomeres and embryos**. Individual embryos or blastomeres at the 8-cell stage were processed in a single tube from collection to template quantification. In order to measure both RNA and DNA copy numbers, RT was carried out in all these samples, followed by duplex LATE-PCR. *Xist*-specific fluorescence (solid lines) is reported on the left scale of each graph, *Oct4*-specific fluorescence (broken lines) is reported on the right scale of each graph. (A) Comparison of *Oct4/Xist *RNA + DNA analysis in a female and a male blastomere. The male blastomere contained a single copy of *Xist *genomic DNA (no Xist RNA), shown by the solid blue line, and a much higher number of *Oct4 *templates (indicating *Oct4 *expression) shown by the broken blue line. The single-copy *Oct4 *signal generated by a polar body is shown for reference (broken green line). The female blastomere also contained numerous *Oct4 *templates (broken red line) and an even higher number of *Xist *templates (solid red line) due to active *Xist *expression. The sex of these blastomeres was confirmed by analysis of the other cells comprising each embryo. (B) Consistency of template measurements in single cells and embryos. The *Xist *signal of an 8-cell male embryo (solid black line) crossed the threshold three duplication cycles earlier than the *Xist *signal of one male blastomere (solid blue line), consistent with the presence of eight genomes in the embryo as also confirmed by *Xist *amplification in 10-genome equivalents of standard DNA (light-green line). In this example, the *Oct4 *template numbers of the cell and embryo analyzed (blue and black broken lines, respectively) showed a 1:8 ratio similar to that of the *Xist *templates. This ratio, however, can vary because, while *Xist *is not expressed in males, *Oct4 *measurements include RNA levels that differ among blastomeres (see Fig. 5).

A RT step was introduced before PCR to measure total (cDNA + genomic DNA) *Oct4*/*Xist *templates in embryos or blastomeres. Female embryos were easily identified by the presence of *Xist *transcripts in their blastomeres; male embryos were characterized by the absence of *Xist *RNA from all cells comprising the embryo. Several observations confirmed that quantification of *Oct4 *and *Xist *copy numbers was accurate. For instance, as expected single male blastomeres contained only one *Xist *template, a single copy of genomic DNA on their X chromosome (see Fig. 4A, solid blue line), while single female blastomeres contained multiple *Xist *templates due to the presence of *Xist *RNA, as indicated by their lower C_T _values (see Fig. 4A, solid red line). Conversely, blastomeres of both sexes contained *Oct4 *RNA and their signals (red and blue broken lines in Fig. 4A) arose several cycles earlier than that of a single copy of *Oct4 *amplified from a polar body (green broken line in the same graph; polar bodies are haploid cells with silent genes). The single-copy *Xist *plots generated by male cells consistently arose about one cycle earlier than the single-copy *Oct4 *signal of the polar body. This result is in agreement with the fact that *Oct4 *and *Xist *signals had similar C_T _values in genomic standards built with male DNA containing one copy of *Xist *and two copies of *Oct4 *per genome (Fig. 3A). Furthermore, the *Xist *signals obtained from whole male 8-cell embryos had C_T _values about three cycles lower than the signal generated by single male cells (compare the solid black line to the solid blue line in Fig. 4B). This delta corresponds to the expected 8-fold increment in template numbers (one duplication at every cycle). Because male embryos do not express *Xist*, they contain only eight copies of *Xist *genomic DNA; the data from these biological samples was, thus, also useful to confirm the reliability of our standards (see the *Xist *10-genome green line in Fig. 4B). The broken lines in Fig. 4B are the co-amplified *Oct4 *plots of the samples analyzed; their shifts compared to the corresponding *Xist *plots prove that *Oct4 *RNA was present and that the RT reaction had worked efficiently.

**Figure 5:**
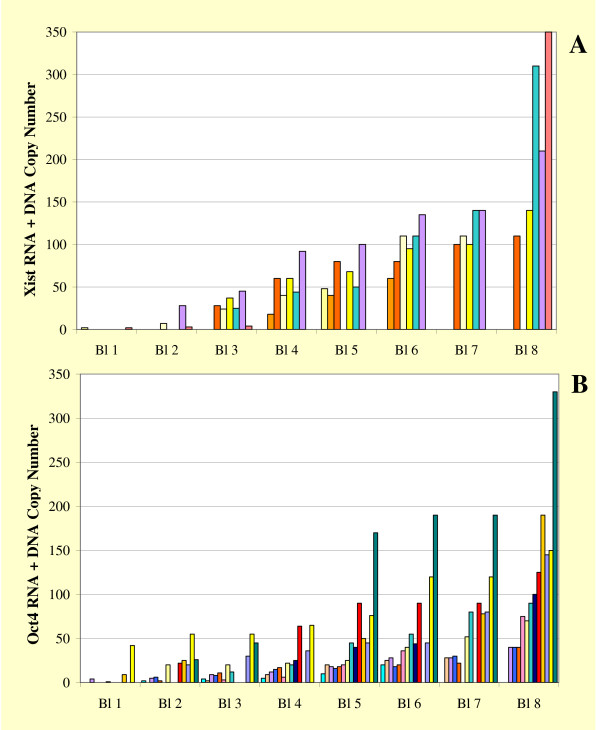
**Variability of *Xist *and *Oct4 *RNA levels among blastomeres of the same 8-cell embryo**. (A) *Xist *RNA+DNA copy numbers measured in the single blastomeres recovered from eight female embryos at the 8-cell stage. Genomic *Xist *DNA amounts to two copies in each cell and is negligible on the scale used for this graph. Bars of the same color indicate cells extracted from the same embryo, which were arranged and numbered in order of increasing *Xist *RNA content. When fewer than eight cells were recovered, the collected blastomeres were assigned to the groups with the closest *Xist *RNA contents. (B) *Oct4 *RNA+DNA levels in individual blastomeres recovered from fourteen male and female 8-cell embryos. Two copies of *Oct4 *template per cell are due to the presence of genomic DNA (negligible on the scale used). Bars were arranged with the same criteria used for *Xist *expression analysis in panel A; colors were used independently in the two panels. By the 8-cell stage blastomeres comprising the same embryo consistently exhibit different levels of expression of both the *Xist *and the *Oct4 *gene.

Analysis of sets of blastomeres isolated from 8-cell embryos clearly demonstrated that both *Xist *and *Oct4 *are expressed to variable degrees in the cells of the same embryo. Figure 5 shows that, for each embryo processed (identified by a given color in each panel), the recovered cells can consistently be arranged according to a gradient of *Xist *or *Oct4 *RNA content (Fig. 5A and 5B, respectively). The mean *Oct4 *RNA content of our samples was lower than the mean *Xist *RNA content (48.7 ± 54.0 *Oct4 *DNA + RNA copies per recovered cell, average ± s.d. of 91 blastomeres, vs 75.1 ± 75.3 *Xist *DNA + RNA copies per recovered cell, average ± s.d. of 43 blastomeres). *Oct4 *RNA levels were not significantly different when calculated separately for female and male embryos in order to allow a better comparison to *Xist *measurements, which included only female blastomeres (49.4 ± 60.2 *Oct4 *DNA + RNA copies per recovered male cell vs 47.3 ± 41.2 *Oct4 *DNA + RNA copies per recovered female cell).

**Figure 6:**
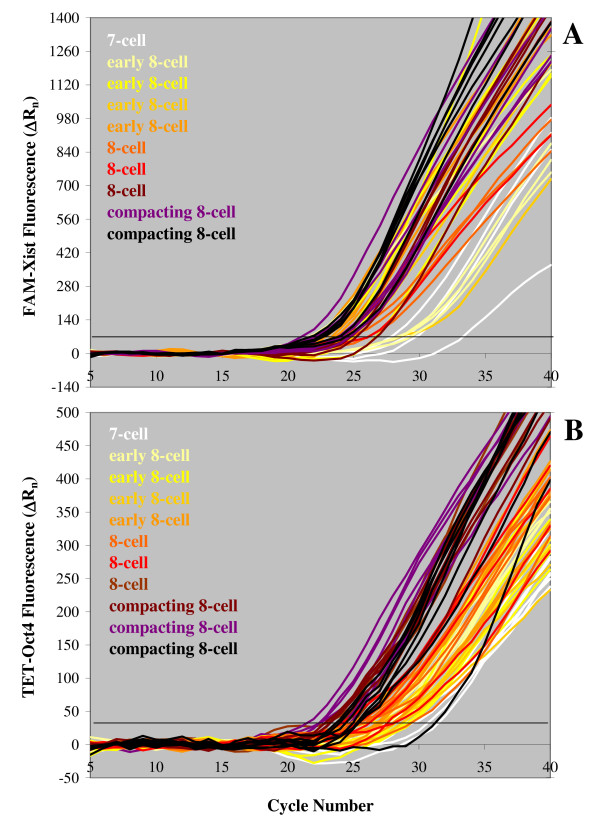
**Increasing *Xist *and *Oct4 *expression in blastomeres of embryos progressing through the 8-cell stage**. (A) *Xist *RNA+DNA amplification plots of single blastomeres from female embryos at the 7-cell to compacting 8-cell stages. A different color was used for each embryo analyzed; plots of cells from the same embryo are shown in the same color. Embryos were labeled as "early 8-cell" when they contained blastomere pairs still connected after cell division. They were identified as "8-cell" when cells were not connected and had well-defined boundaries, and as "compacting 8-cell" when compaction had begun and blastomere counts were finalized only after de-compaction and cell harvesting. Progression through the 8-cell stage is visualized by increasingly darker hues. Within each group, embryos slower to reach the indicated stage were assigned a lighter color than faster-developing embryos. (B) *Oct4 *RNA+DNA real-time PCR curves generated by single blastomeres of male and female embryos at the third cleavage stage. Color-coding followed the same criteria used for the *Xist *plots in panel A, but colors were used independently in the two panels. Although cells from each embryo consistently produced signals spread over several PCR cycles, overall *Xist *and *Oct4 *expression tended to increase in embryos approaching compaction, as shown by the right-to-left shift of the plots.

**Figure 7:**
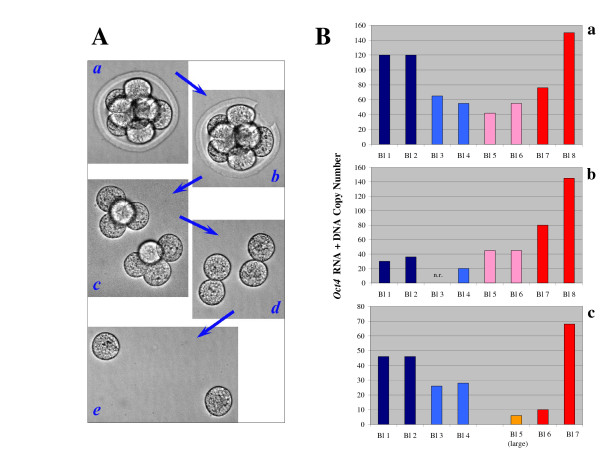
**Distribution of *Oct4 *RNA in newly-divided blastomeres of 8-cell embryos**. (A) The zona pellucida of embryos that had just reached the 8-cell stage (*a*) was perforated with three laser pulses (*b*). Gentle pipetting resulted in the extrusion of two groups of four connected cells from the zona (*c*). Each quartet was divided in two cell pairs (*d*) and, finally, in single blastomeres (*e*) by further up-and-down pipetting. (B) *Oct4 *RNA+DNA levels in the cells recovered from two embryos that had just reached the 8-cell stage (*a *and *b*) and one 7-cell embryo (*c*). All cells but one (n.r.) were recovered. *Oct4 *template measurements in cells deriving from pairs are shown by bars of the same color; the same hue (bluish or reddish) indicates cells originally forming a quartet. The 7-cell embryo was comprised by a quartet and a triplet of cells. *Oct4 *RNA+DNA levels in the largest cell of the triplet are shown by the orange bar (Bl. 5 in *c*). *Oct4 *RNA copy numbers were similar in the two cells of newly-formed pairs, with the exception of one higher-expressing blastomere per embryo.

Total *Xist *and *Oct4 *RNA content per embryo varied substantially, even considering that not all cells were recovered from each specimen, although *Xist *RNA levels fell in the range that we have reported previously using symmetric PCR [4]. To some extent, such variability was linked to the embryos' developmental progress, as illustrated by the real-time PCR plots in Fig. 6 (6A: *Xist*; 6B: *Oct4*). The fluorescent signals in these charts were generated by single blastomeres from a 7-cell embryo and from 8-cell embryos that: I) had just reached this stage ("early 8-cell," containing pairs of cells still connected by a cytoplasmic bridge, see also Fig. 7), or II) contained completely separated cells with clearly identifiable boundaries ("8-cell"), or III) were compacting (embryos de-compacted once placed in the calcium-free medium used for blastomere isolation and cells could be counted). Expression of both genes under study generally increased as embryos progressed through the 8-cell stage, as shown by the right-to-left shift of the plots of the compacting embryos compared to the early 8-cell embryos. Samples of the three groups, however, overlapped considerably as it could be expected from the data reported in Fig. 5, because each embryo (independently from its developmental progression) contained high- and low-expressing cells. In the case of *Oct4 *RNA, this result was also partly explained by the finding that most female embryos developed more slowly than male embryos of the same group, as already described by other authors [36-38]. Quantification of *Oct4 *RNA levels in cells from embryos at the 7- to very early 8-cell stage indicated that *Oct4 *expression is initially similar in pairs of daughter cells. In the experiment summarized in Fig. 7, three embryos were processed soon after they had passed the 6-cell stage. Two of the embryos were comprised by eight cells and were easily separated in two quartets upon zona drilling and gentle pipetting (Fig. 7A, a, b, c). Further and longer pipetting of the quartets produced four pairs of blastomeres (Fig. 7A, d), which were in turn divided into eight single blastomeres (Fig. 7A, e). The third embryo analyzed was a 7-cell embryo which was disassembled in similar manner, except that the first separation produced one quartet and one triplet including a cell visibly bigger than the others. Bar graphs of the same color in Fig. 7B show the *Oct4 *RNA + DNA content of cells from pairs; bar graphs of the same hue (bluish or reddish) represent results obtained from cell quartets (or from the triplet of the 7-cell embryo, reddish hue in Fig. 7B, c). In all instances, *Oct4 *RNA levels were similar in cells of the same pair, with the exception of one pair in each embryo. It is possible that the pair of blastomeres containing different amounts of *Oct4 *transcripts was the first among the four to divide and that differences in *Oct4 *RNA content between sister cells were being established as the cell cycle progressed.

### Correlation analysis of *Oct4 *and *Xist *RNA levels in series of blastomeres of female 8-cell embryos

**Figure 8:**
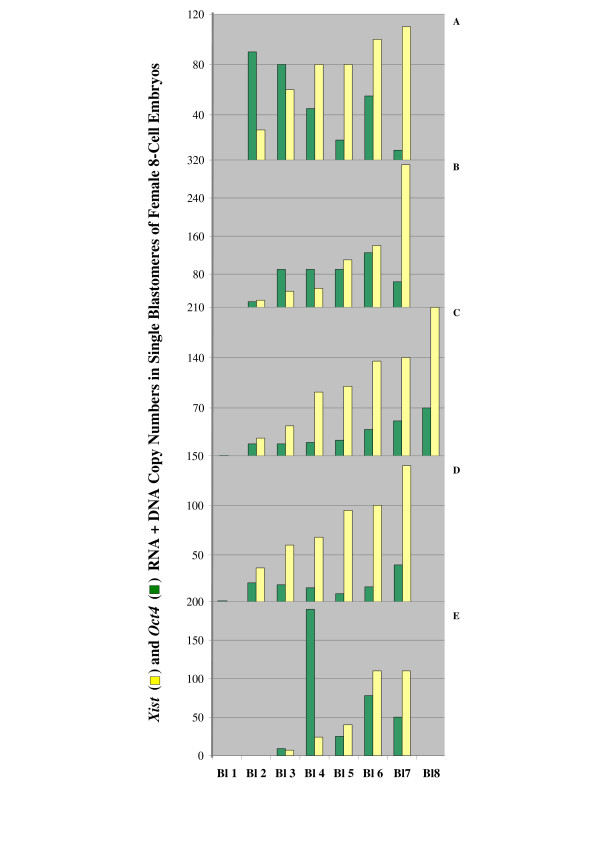
**Duplex quantification of *Oct4*/*Xist *transcripts in series of single blastomeres from female 8-cell embryos**. *Xist *and *Oct4 *RNA+DNA copy numbers measured in all cells recovered from five female 8-cell embryos (A to E). Cells were arranged and numbered based on increasing *Xist *template content (yellow bars). The respective *Oct4 *template content of each cell is shown by the green bar adjoining the yellow bar. Relative *Oct4 *and *Xist *expression patterns were variable from embryo to embryo, but specific cells contained predominantly one of the two transcripts.

Although the 8-cell embryos analyzed in this work were comprised by cells with different amounts of *Oct4 *and *Xist *RNA (Figs. 5 and 6), we did not find at this point in development a consistent correlation, either positive or negative, in the expression levels of these two genes in individual blastomeres of the same embryo. Figure 8 shows the results obtained from the quantification of *Oct4 *and *Xist *RNA (variable) + DNA (a constant of two copies per cell) copy numbers in the blastomeres recovered from five female embryos. In each graph the recovered cells are arranged in order of increasing *Xist *template contents (yellow bars) and the *Oct4 *template contents of the same cells are shown by the adjacent green bars. Only in one case (Fig. 8A) did we observe an overall reciprocal *Oct4*/*Xist *expression pattern which included all cells recovered from that embryo, so that blastomeres containing more *Xist *transcripts had gradually less *Oct4 *RNA and vice versa. In the other embryos *Oct4 *and *Xist *expression levels per cell appear to be unrelated. However, strongly reversed expression was observed in specific blastomeres (see Bl. 7 of Embryo B for high *Xist *transcription and low *Oct4 *transcription, and Bl. 4 of Embryo E for the opposite case), suggesting the possibility that in these embryos establishment of differential patterns was in an initial phase and restricted to some cells.

## Discussion and Conclusion

Investigators trying to develop assays for the accurate multiplex quantification of gene expression in small samples face many technical hurdles [5]. We report here that the combination of single-tube PurAmp sample preparation and LATE-PCR offers a new and effective strategy to overcome these problems. It is now possible to simultaneously generate multiple amplicons with optimized efficiency, even when the initial numbers of target sequences differ by several orders of magnitude.

*Oct4 *RNA is present in all cleavage-stage blastomeres, while *Xist *RNA is present in hundreds of copies in female cells but is absent from male cells. The simultaneous measurement of these two transcripts in embryos of both sexes presents, therefore, a challenging situation, because it requires that one amplicon (*Oct4*) is reliably generated with equal efficiency and sensitivity in the absence as well as in the presence of high amounts of a second amplicon (*Xist*). In our study, *Oct4 *amplification was not affected by *Xist *co-amplification, as established by careful analysis of the slopes of the real-time plots generated by different concentrations of each template, a very sensitive diagnostic parameter of LATE-PCR. Furthermore, no significant differences were observed in the *Oct4 *RNA contents of male and female embryos, independently from the absence or presence of *Xist *RNA and even though, on average, male embryos develop to the 8-cell stage faster than female embryos [36-38]. Thus, LATE-PCR offers a highly advantageous alternative to other quantitative PCR-based strategies that require prior knowledge of the relative concentration of the different templates to be amplified and, hence, are suited to multiplexing of internal standards but cannot be utilized for unknown samples [5].

In addition, the plots generated during real-time LATE-PCR are linear and, therefore, the fluorescence intensities reached after a given number of cycles are proportional to the number of target sequences present at the beginning of the reaction. This feature of LATE-PCR and RT-LATE-PCR opens up the possibility of end-point quantification of multiple amplicons. Preliminary experiments carried out in this laboratory have already demonstrated the validity of this approach and we envision the future application of this technique to gene expression quantification.

The present study proves that cells comprising the same embryos contain markedly dissimilar levels of both *Oct4 *and *Xist *transcripts, an indication that 8-cell blastomeres are not equivalent in their developmental properties, in agreement with the hypothesis that lineage commitment in the mammalian embryo starts very early [39] and possibly at the time of fertilization [14]. Quantification of differential expression of genes involved in development contributes to this hypothesis at a deeper level than possible with traditional non-quantitative techniques, such as fluorescence in situ hybridization (FISH), agarose gel electrophoresis and immunofluorescence. For example, gel analysis suggests that the presence of *Oct4 *RNA in human 8-cell embryos is limited to a subset of blastomeres [27], while this work demonstrates that each embryo is comprised by an assortment of low-to-high expressing cells. Although we cannot exclude species-specific differences, our findings support a "gradient" model of gene activation that can be reconciled with the well-known regulative capacity of the mammalian embryo [14,40] more easily than an "on/off" model. In addition, analysis of newly-formed blastomeres, identified through two division cycles based on their surviving connections [14], reveals that these paired cells contain similar numbers of *Oct4 *transcripts, a situation comparable to the equal OCT4 status seen in daughter cell pairs of human embryonic stem cells [41]. These data indicate that differences in *Oct4 *expression among 8-cell embryonic blastomeres are established sometimes after cell division and are probably regulated by a number of unknown factors. It is possible that cells that most actively transcribe *Oct4 *are initially fated to be the founders of the pluripotent ICM and germ cell lineages [42]. In the case of stress, death or removal of these cells for genetic testing, however, *Oct4 *expression in other blastomeres could be up-regulated to levels adequate for the formation of a new pluripotent lineage, thus preserving the integrity of the developing embryo. A similar regulation pathway can be envisioned for the correct expression of *Xist *in the TE lineage, although the 8-cell stage may be too early for the full establishment of complementary expression patterns of these two genes in all cells comprising an embryo. It should also be kept in mind that, in spite of its advantages for monitoring embryonic development, the use of frozen-thawed or cultured embryos rather than ex vivo specimens may lead to some alteration in gene expression patterns, even though a recent, large-scale study indicates that expression levels at the 8-cell stage are almost unaffected by culture in vitro [43]. (These data were obtained with mouse embryos and may differ for other species.)

Beyond the details of our particular experimental model, the achievement of reliably quantitative multiplex measurements of gene expression in single cells is meaningful for a wide variety of applications. For instance, the ability to identify blastomeres with specific developmental potential at the earliest possible stage would have great relevance for embryonic stem cells preparation [44,45]. Quantification of the activation of *Oct4 *and other genes involved in maintaining pluripotency is a step necessary to reach this goal, as demonstrated by an increasing number of reports [23,41,46]. On the other hand, the advancing knowledge that 8-cell blastomeres are not developmentally equivalent as assumed in the past also carries implications for the practice of preimplantation genetic diagnostics, which requires the random resection of at least one blastomere from human embryos [40]. Testing expression levels of specific genes in the ablated blastomeres may, however, offset the disadvantages of the procedure by providing additional information on the embryo's health, assuming that analysis of one blastomere was reliably representative for the entire embryo. Preliminary experiments in our laboratory indicate, for example, that transcription of the stress chaperone *hsp70 *is up-regulated in slow-growing embryos (Anshelevich A. et al., unpublished observations). A similar increased expression of heat shock protein 70 also occurs in microsatellite-unstable colorectal cancers and is correlated with prognosis [47]. On a larger scale, epigenetic analyses of normal and tumor cells have recently revealed a wide array of changes in genetic activity in cells that have undergone cancerous transformation [48]. Intensive work is under way to identify which of those changes could be most valuable for use as diagnostic tools. Improvements in the methods of multiplexed quantification of gene expression thus hold great promise also for this emerging field of studies, particularly when such methods are suitable for small-sample or single-cell analysis as the one described in this work.

## Methods

### Embryo culture and single blastomere collection

Embryos from B6C3F1 female mice bred with B6D2F1male mice were purchased frozen at the two-cell stage from Embryotech Laboratories, Inc. (Wilmington, MA) and cultured in GEM-PS medium (Duncan Holly, Bedford, MA) to the desired developmental stage, as described elsewhere [3,4]. For most experiments, series of single blastomeres were harvested from individual 8-cell embryos following laser-zona drilling with a noncontact 1,480-nm diode laser beam (ZILOS-tkTM zona infrared laser optical system; Hamilton Thorne Biosciences, Inc., Beverly, MA). We have previously demonstrated the safety and reliability of this method for RNA recovery [3,4,11].

Single trophectodermal cells or small groups of TE cells were obtained from blastocysts that had just begun spontaneous hatching (no laser was used in this case). Repeated pipetting of these embryos up-and-down produced the release of individual cells that were protruding outside the zona. Only spherical, undamaged cells were used for gene expression analyses.

### ICM isolation by immunosurgery

ICMs were prepared from early blastocysts as detailed by Eckert *et al.*[49] with some modifications. The zona pellucida was removed with Acid Tyrode's Solution (Sigma Chemical Company, St. Louis, MO), pH 2.3, in glass bottom dishes. Blastocysts were allowed to recover for 20 min in Modified Human Tubal Fluid (HTF) Medium, Hepes-buffered (Irvine Scientific, Santa Ana, CA) containing 1 mg/ml embryo-grade bovine serum albumin (Sigma) (HTF-BSA) at 37°C. They were then incubated for 10 min at room temperature in 200 μl of a 5% (w/v) solution of Trinitrobenzene sulphonic acid (TNBS; Sigma) diluted 1:10 with HTF containing 0.4% polyvinyl pyrrolidone (HTF-PVP), pH 7.4. After three washes in HTF-PVP, embryos were incubated for 10 min at room temperature in 25 μl droplets of goat antidinitrophenyl BSA (anti-DNP BSA; ICN Biochemicals, now MP Biochemicals, Irvine, CA) diluted in HTF-PVP (0.1 mg/ml anti-DNP BSA final concentration), under oil. Samples were again washed three times in HTF-PVP and incubated at 37°C for 10 min in 25 μl-droplets under oil containing guinea pig complement. (Guinea Pig Complement Serum reconstituted in ice-cold water and diluted 1:10 in cold HTF-BSA; Sigma.) Following a prolonged wash (at least 20 min at 37°C) in HTF-BSA, the ICMs were shelled out by repeated pipetting through a microcapillary polished with a microforge. Cell debris was removed by a final rinse HTF-BSA.

### Single-tube sample collection and reverse transcription

Single embryos, single blastomeres or ICMs were delivered to dry droplets of denaturing reagents placed on PCR tube lids (LysoDots), briefly heated and dried again and stored at -20°C, as detailed in reference [3]. A mixture of Random Hexamers (1.5 μl of a 50 ng/μl stock solution) in DEPC-treated water (4.5 μl) was then added to each lysed sample directly into the lid. (All RT reagents were from a ThermoScript™ RT-PCR System kit, Invitrogen, Life Technologies, Carlsbad, CA.) The sample was spun down and the remaining RT reagents were delivered to the same tube, according to the PurAmp procedure. As previously described [3,10], all RT reagents were used at the suggested concentrations except for the absence of DTT, but volumes were halved so that each assay was performed in just 10 μl, which increased to 10.5 μl after RNase H digestion.

At the end of RT, real-time PCR was carried out by adding the appropriate reagents still in the same vessel, as described below.

### Duplex LATE-PCR assay for *Oct4*/*Xist *RNA and DNA measurements in individual embryos or blastomeres

We designed the duplex real-time LATE-PCR assay for simultaneous amplification of sequences within exons of the murine *Oct4 *and *Xist *genes (GenBank Accession Number NM_013633, *Oct4 *mRNA, and 202420, *Xist *mRNA). (Because the PCR primers used for this work are not strand-specific, transcripts of the antisense gene *Tsix *could also be amplified during *Xist *PCR [4]. However, *Tsix *expression in mouse embryos starts after the 8-cell stage [50].) Each reaction was run in a final volume of 50 μl and contained the following reagents: 1x PCR buffer (Invitrogen) comprised by 20 mM Tris-HCl, pH 8.4, and 50 mM KCl, 3 mM MgCl_2_, 0.4 mM of each dNTP, 50 nM *Oct4 *Limiting Primer having the sequence 5' TGGCTGGACACCTGGCTTCAGACT 3', 2 μM *Oct4 *Excess Primer having the sequence 5' CAACTTGGGGGACTAGGC 3', 100 nM *Xist *Limiting Primer having the sequence 5' GGTCGTACAGGAAAAGATGGCGGCTCAA 3', 2 μM *Xist *Excess Primer having the sequence 5' TGAAAGAAACCACTAGAGGGCA 3', 1 μM *Oct4 *molecular beacon having the sequence 5' TET-CCG CCT GGG ATG GCA TAC TGT GGA AGG CGG-Dabcyl 3', 1 μM *Xist *molecular beacon having the sequence 5' FAM-CGT GGG TGT CTA AGA TGG CGG AAG TCC CAC G-Dabcyl 3', 0.3× PrimeSafe™ -043 (a mis-priming-preventing compound available from Smiths Detection at biodetection@smithsdetection.com, [51]), and 2 units of antibody-complexed Platinum^® ^Taq DNA polymerase (Invitrogen, Carlsbad, CA). Molecular beacons were obtained from Integrated DNA Technologies (Coralville, IA).

(A simplified version of this assay was employed for the initial measurements of *Oct4 *templates only. All components were the same, except for the absence of *Xist *primers and probe and the fact that 0.3× PrimeSafe-045 was used in this case. The concentration of dNTPs was decreased to 0.25 mM and that of Taq DNA polymerase to 1 unit.)

PCR was carried out in an ABI Prism 7700 Sequence Detector (Applied Biosystems, CA) with a thermal profile comprised by 1 cycle at 95°C for 5 minutes; 15 cycles at 95°C for 10 sec, 63°C for 20 sec, and 72°C for 30 sec; and 40 cycles at 95°C for 15 sec, 55°C for 25 sec, 72°C for 35 sec, and 45°C for 30 sec, with fluorescence acquisition at 45°C in the TET and FAM channels. At the end of the reaction, the trendline of each fluorescent signal was plotted with Microsoft Excel software (moving average with period 4).

### Amplicon identification and sequencing

Amplification products were analyzed by gel electrophoresis in a 3% agarose gel in 0.5× TBE buffer for 2 hours and stained with ethidium bromide. Both the double-stranded and the single-stranded products (more weakly stained), of the expected sizes, were visible for the two co-amplified genes, indicating efficient duplex LATE-PCR.

The identity of the amplification products obtained from *Oct4 *LATE-PCR and RT-LATE-PCR was also confirmed by direct dideoxysequencing after a simple dilution step. No purification was needed because the overwhelming majority of the amplicons generated by LATE-PCR is single-stranded ("Dilute'N Go" sequencing) [9,51]. Analysis of the sequences was carried out using Chromas software (Technelysium Pty Ltd).

### Template quantification

Male mouse genomic DNA (The Jackson Laboratory, Bar Harbor, ME) was utilized to prepare standard curves for quantification of *Oct4 *and *Xist *templates based on known genome numbers [4]. Using these curves, the "threshold cycle" (C_T_) at which the fluorescent signal of an unknown sample is first detected above background can be converted into a copy number [6]. Under optimal conditions, a two-fold difference in the number of templates amplified results in a shift of one cycle between two C_T _determinations. A more abundant template has a lower C_T _value than a less abundant template, because it accumulates sooner to a detectable level [6]. One male mouse genome contains one copy of *Xist *(X-chromosome) and two copies of *Oct4 *(chromosomes 17).

## Authors' contributions

CH designed the assay, conducted single-blastomere quantification experiments and drafted the manuscript. JJE performed the immunosurgery experiment and shared her mouse embryo expertise. OH contributed to the quantitative analyses and to the optimization of the PCR assay with PrimeSafe. LJW coordinated the study and the exchanges between the two laboratories involved.
